# Folding and faulting of an elastic continuum

**DOI:** 10.1098/rspa.2016.0018

**Published:** 2016-03

**Authors:** Davide Bigoni, Panos A. Gourgiotis

**Affiliations:** DICAM, University of Trento, via Mesiano 77, 38123 Trento, Italy

**Keywords:** Cosserat elasticity, ellipticity, stress channelling, dilation/compaction, extreme orthotropy, Green's functions

## Abstract

Folding is a process in which bending is localized at sharp edges separated by almost undeformed elements. This process is rarely encountered in Nature, although some exceptions can be found in unusual layered rock formations (called ‘chevrons’) and seashell patterns (for instance *Lopha cristagalli*). In mechanics, the bending of a three-dimensional elastic solid is common (for example, in bulk wave propagation), but folding is usually not achieved. In this article, the route leading to folding is shown for an elastic solid obeying the couple-stress theory with an extreme anisotropy. This result is obtained with a perturbation technique, which involves the derivation of new two-dimensional Green's functions for applied concentrated force and moment. While the former perturbation reveals folding, the latter shows that a material in an extreme anisotropic state is also prone to a faulting instability, in which a displacement step of finite size emerges. Another failure mechanism, namely the formation of dilation/compaction bands, is also highlighted. Finally, a geophysical application to the mechanics of chevron formation shows how the proposed approach may explain the formation of natural structures.

## 1. Introduction

**Figure 1.**
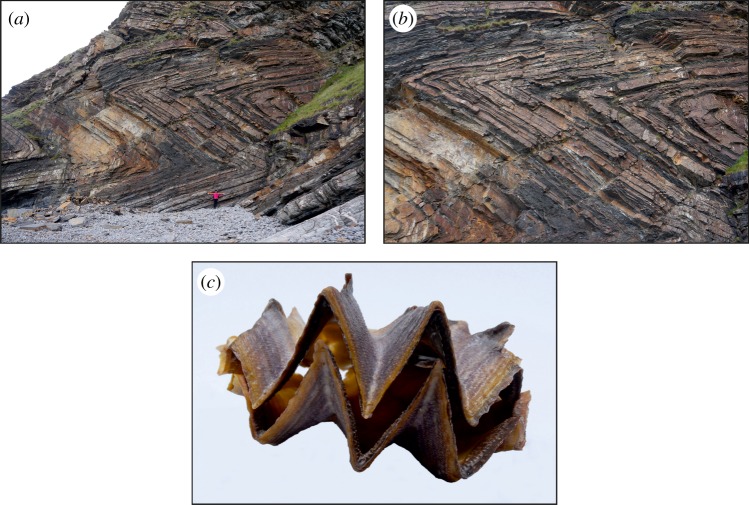
(*a*) Chevron folds in layered rocks near Millook Haven (UK). (*b*) Detail of the chevron folding. (*c*) A *Lopha cristagalli* seashell, also called a ’cockscomb oyster’, is common in the Indo-West Pacific, where it lives in coral reefs at shallow depth. The shell clearly shows folding. (Online version in colour.)

Examples of folding in Nature, where almost undeformed layers terminate with narrow zones of exceptionally high curvature, are rare, but they do exist, as demonstrated by chevron rock formations (geological structures characterized by localized folded beds with sharp hinges (figure 1*a*,*b*; see also [1])) and some seashells (for instance, *Lopha cristagalli* (figure 1*c*) or *Dendostrea folium*). Folding is an extremely localized bending process and cannot be modelled within the framework of the linear theory of (Cauchy) elasticity,^1^ while Cosserat elasticity has already been advocated as particularly suited to model layered rock and fibre-reinforced materials [7–11].

**Figure 2.**
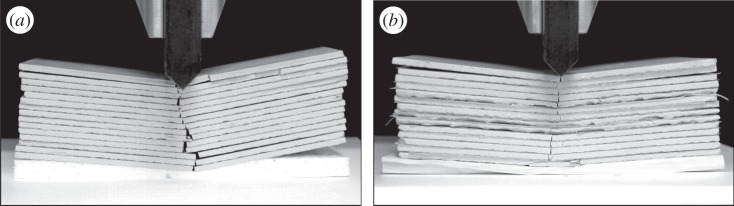
A stack of unglazed ceramic tiles (simply piled on top of each other in (*a*) and intercalated with lattice layers 0.3 mm thick in (*b*)) were fractured using a vertical wedge-shaped stainless indentor in a quasi-static test (a Beta 100 electromechanical testing machine from Messphysik was used). The photographs provide an intuitive explanation of how a folded structure can result from a stack of layers, each behaving as a plate which is broken with a localized bending eventually resulting in a crack.

As figure 1 reveals, folding is deeply connected to the layered nature of the material. Indeed, figure 2 shows photographs of a stack of tiles (simply piled on top of each other in figure 2*a* and intercalated with 0.3 mm thick lattice layers in figure 2*b*^2^), fractured by a vertical wedge-shaped stainless indentor and evidencing the formation of a folded structure. The structure is the result of localized bending under the indentor, which becomes a crack due to the brittleness of the material.

Recently, Gourgiotis & Bigoni [12,13] demonstrated that folding can be explained within the constrained Cosserat elasticity with extreme orthotropy. Their results, however, were confined to the case of antiplane strain. The aim of this article is to extend our study to the richer (and more complex) framework of two-dimensional plane-strain-constrained Cosserat elasticity. The constrained Cosserat theory is chosen here since it is the simplest gradient-type generalized continuum theory involving couple-stresses that enriches the classical continuum with additional material characteristic lengths (for a review, see [14]). In this context, two new Green's functions are derived (for applied concentrated force and concentrated bending moment; §3) and employed as perturbations revealing the behaviour of the material when close to a loss of ellipticity (E). It is shown that, near this instability threshold, in-plane folding occurs in a similar manner to that shown in figures 1 and 2. Moreover, the material is also shown to be prone to faulting, when a concentrated moment is applied. It should be noted that Green's functions are to be understood as perturbations, so that they need not exist in reality, but demonstrate that the material tends towards states of folding and faulting when subject to mechanical actions. In other words, folding and faulting seem to emerge as material instabilities for a constrained Cosserat anisotropic material, similar to the situation occurring when a shear band forms in an elastoplastic material^3^ [15]. It should finally be mentioned that, in addition to folding and faulting, a mode of instability in dilation/compaction bands is found at (E) loss (§7). This instability, manifesting itself with a localized increase/decrease of volumetric strain (found, for instance, in sand and sandstone [16]), is found to be connected to the fact that *P*-waves propagate in a constrained Cosserat material as in a Cauchy continuum, so that this failure mode is driven by the loss of (E) of the underlying Cauchy material.

A specific geophysical application to chevron formation in layered rock (§8) shows that the presented model provides a possible explanation of folding in natural materials. Finally, the presented results introduce the possibility of realizing artificial materials with extreme stiffness contrast which can display origami-pattern deformations.

## 2. Basic equations in plane strain

The linearized plane-strain theory of couple-stress elasticity is introduced for homogeneous orthotropic elastic solids. Detailed presentations of the couple-stress theory for isotropic materials can be found in [17,18]. Recently, Gourgiotis & Bigoni [12] examined anisotropic couple-stress solids and provided the conditions of strong ellipticity (SE), ellipticity (E) and wave propagation (WP) that were used as stability criteria in order to study stress channelling and related localization phenomena in extreme constrained Cosserat materials; these will be briefly contextualized here.

### (a) The mechanics of an orthotropic material under plane-strain conditions

When plane-strain conditions prevail, the displacement field can be expressed in the (*x*_1_,*x*_2_)-plane as

2.1 $$u_{1}\equiv u_{1}(x_{1},x_{2})\;\text{and}\;u_{2}\equiv u_{2}(x_{1},x_{2}),$$

while the out-of-plane component of the displacement is null.

The non-vanishing strain, rotation and curvature components are given in the forms

2.2 $$\begin{array}{rl} \varepsilon_{11} & =\frac{\partial u_{1}}{\partial x_{1}},\;\varepsilon_{22}=\frac{\partial u_{2}}{\partial x_{2}},\;\varepsilon_{12}=\varepsilon_{21}=\frac{1}{2}\left(\frac{\partial u_{2}}{\partial x_{1}}+\frac{\partial u_{1}}{\partial x_{2}}\right),\\ \omega_{3} & =\frac{1}{2}\left(\frac{\partial u_{2}}{\partial x_{1}}-\frac{\partial u_{1}}{\partial x_{2}}\right),\;\kappa_{13}=\frac{\partial \omega_{3}}{\partial x_{1}},\;\kappa_{23}=\frac{\partial \omega_{3}}{\partial x_{2}}. \end{array}\}$$

The equations of equilibrium can be written as

2.3 $$\begin{array}{r} \frac{\partial \sigma_{11}}{\partial x_{1}}+\frac{\partial \sigma_{21}}{\partial x_{2}}+X_{1}=0,\;\frac{\partial \sigma_{12}}{\partial x_{1}}+\frac{\partial \sigma_{22}}{\partial x_{2}}+X_{2}=0\;\text{and}\;\sigma_{12}-\sigma_{21}+\frac{\partial m_{13}}{\partial x_{1}}+\frac{\partial m_{23}}{\partial x_{2}}+Y_{3}=0, \end{array}$$

where (*σ*_11_,*σ*_12_,*σ*_21_,*σ*_22_) and (*m*_13_,*m*_23_) are the non-vanishing components of the (asymmetric) stress and couple-stress tensors, respectively. In addition, (*X*_1_,*X*_2_) denote the in-plane components of the body force and *Y*_3_ the out-of-plane component of the body moment.

Further, considering an orthotropic *centrosymmetric* material, whose principle axes are aligned with the Cartesian axes, the strain energy density reduces to [19]

2.4 $$\begin{array}{rlr} W & =\frac{1}{2}\varepsilon \cdot C[\varepsilon ]+\frac{1}{2}\kappa \cdot B[\kappa ] & =\frac{1}{2}\left(c_{11}\varepsilon_{11}^{2}+c_{22}\varepsilon_{22}^{2}\right)+c_{12}\varepsilon_{11}\varepsilon_{22}+2c_{66}\varepsilon_{12}^{2}+2\eta_{1}\kappa_{13}^{2}+2\eta_{2}\kappa_{23}^{2}, \end{array}$$

with the fourth-order elasticity tensors C and B defined as

2.5 $$C=c_{11}{\text{M}}_{1}⊗{\text{M}}_{1}+c_{22}{\text{M}}_{2}⊗{\text{M}}_{2}+c_{12}({\text{M}}_{1}⊗{\text{M}}_{2}+{\text{M}}_{2}⊗{\text{M}}_{1})+2c_{66}({\text{M}}_{1}\;\underline{\bar{⊗}}\;{\text{M}}_{2}+{\text{M}}_{2}\;\underline{\bar{⊗}}\;{\text{M}}_{1})$$

and

2.6 $$B=8\eta_{1}\left({\text{M}}_{1}\;\underline{\bar{⊗}}\;{\text{M}}_{3}+{\text{M}}_{3}\;\underline{\bar{⊗}}\;{\text{M}}_{1}\right)+8\eta_{2}\left({\text{M}}_{2}\;\underline{\bar{⊗}}\;{\text{M}}_{3}+{\text{M}}_{3}\underline{\bar{⊗}}{\text{M}}_{2}\right),$$

where **e**_*i*_ (*i*=1,2,3) is an orthonormal basis, **M**_*i*_=**e**_*i*_⊗**e**_*i*_, and the tensorial product $\underline{\bar{⊗}}$ is defined as ${\left(\text{A}\;\underline{\bar{⊗}}\;\text{B}\right)}_{ijkh}=1/2(A_{ih}B_{jk}+A_{ik}B_{jh}),\;\forall A_{ij},B_{ij}$.

Accordingly, the following constitutive equations can be derived for the components of the symmetric part of the stress tensor *τ*_*ij*_=*σ*_(*ij*)_ and the couple-stress tensor, respectively:

2.7 $$\tau_{11}=c_{11}\varepsilon_{11}+c_{12}\varepsilon_{22},\;\tau_{22}=c_{12}\varepsilon_{11}+c_{22}\varepsilon_{22}\;\text{and}\;\tau_{12}=\tau_{21}=2c_{66}\varepsilon_{12}$$

and

2.8 $$m_{13}=4\eta_{1}\kappa_{13}\;\text{and}\;m_{23}=4\eta_{2}\kappa_{23},$$

with *c*_11_, *c*_12_, *c*_22_ and *c*_66_ being the ‘classical’ moduli characterizing the underlying orthotropic Cauchy material subject to plane-strain conditions, and *η*_1_, *η*_2_ the couple-stress bending moduli with the dimension of a force. In addition, in view of equation (2.3)_3_, the components of the antisymmetric part of the stress tensor *α*_*ij*_=*σ*_[*ij*]_ become

2.9 $$\alpha_{11}=\alpha_{22}=0,\;\alpha_{12}=-\alpha_{21}=-2\left(\eta_{1}\frac{\partial^{2}\omega_{3}}{\partial x_{1}^{2}}+\eta_{2}\frac{\partial^{2}\omega_{3}}{\partial x_{2}^{2}}\right)-\frac{1}{2}Y_{3}.$$

The strain energy density *W* is positive definite (PD) when the material moduli satisfy the following inequalities:

2.10 $${\left(\text{PD}\right)}^{C}\Leftrightarrow c_{11}>0,\;c_{22}>0,\;-\sqrt{c_{11}c_{22}}<c_{12}<\sqrt{c_{11}c_{22}},\;c_{66}>0$$

and

2.11 $${\left(\text{PD}\right)}^{B}\Leftrightarrow \eta_{1}>0,\;\eta_{2}>0.$$

Enforcing equilibrium yields a coupled system of PDEs of the fourth order for the in-plane displacement vector **u**=(*u*_1_,*u*_2_), which can be concisely written as

2.12 Lu+F=0,

where the matrix differential operator is defined as

2.13 $$\text{L}={\text{L}}^{C}+{\text{L}}^{P},$$

with

2.14 $${\text{L}}^{C}=\left[\begin{array}{cc} c_{11}\partial_{1}^{2}+c_{66}\partial_{2}^{2} & \left(c_{12}+c_{66}\right)\partial_{1}\partial_{2}\\ \left(c_{12}+c_{66}\right)\partial_{1}\partial_{2} & c_{66}\partial_{1}^{2}+c_{22}\partial_{2}^{2} \end{array}\right]\;\text{and}\;{\text{L}}^{P}=\left(\eta_{1}\partial_{1}^{2}+\eta_{2}\partial_{2}^{2}\right)\left[\begin{array}{cc} -\partial_{2}^{2} & \partial_{1}\partial_{2}\\ \partial_{1}\partial_{2} & -\partial_{1}^{2} \end{array}\right],$$

in which ∂_*q*_≡∂()/∂*x*_*q*_, **L**^*P*^ is the principal operator and **F** is a generalized force with in-plane components

2.15 $$F_{1}=X_{1}+\frac{1}{2}\partial_{2}Y_{3}\;\text{and}\;F_{2}=X_{2}-\frac{1}{2}\partial_{1}Y_{3}.$$

Finally, it is remarked that, when *c*_11_=*c*_22_=λ+2*μ*, *c*_12_=λ, *c*_66_=*μ* and *η*_1_=*η*_2_=*η*, the above equations reduce to those governing *isotropic* couple-stress elasticity for plane-strain deformations [17]. On the other hand, setting *η*_1_=*η*_2_=0 and *Y*_3_=0 (no couple-stress effects), equation (2.12) degenerates to the classical Navier–Cauchy equations of equilibrium.

### (b) Strong ellipticity and the wave propagation condition

The following definitions of (SE) for the elasticity tensors C and B are introduced following Gourgiotis & Bigoni [12]:

2.16 (q⊗n)⋅C[q⊗n]>0and(q⊗n)⋅B[q⊗n]>0,

to be satisfied for every unit vector **n** and **q**. From equations (2.16) and using equations (2.5) and (2.6), it can be readily shown that, in the plane-strain case, the (SE) conditions impose the following constraints for the elastic moduli:

2.17 $${\left(\text{SE}\right)}^{C}\Leftrightarrow c_{11}>0,\;c_{22}>0,\;c_{66}>0,\;-2c_{66}-\sqrt{c_{11}c_{22}}<c_{12}<\sqrt{c_{11}c_{22}}$$

and

2.18 $${\left(\text{SE}\right)}^{B}\Leftrightarrow \eta_{1}>0,\;\eta_{2}>0.$$

It is clear that if the elasticity tensors are (PD) they are also (SE). The importance of the (SE) conditions is that they are sufficient for uniqueness in a problem with prescribed displacement and rotation on the whole boundary (kinematical boundary conditions) for a homogeneous constrained Cosserat solid. This statement represents the extension of van Hove's theorem to the context of the constrained Cosserat theory [12].

For an orthotropic constrained Cosserat medium under plane-strain conditions, the propagation of plane harmonic waves is examined by augmenting the governing equations (2.12) with the inertia term $\rho \ddot{\text{u}}$ (micro-rotational inertia is neglected in this study). Assuming zero body forces and body moments, the equations of motion become

2.19 $$\text{Lu}=\rho \ddot{\text{u}},$$

where *ρ*>0 is the constant mass density and the superposed dot denotes time differentiation.

A plane-wave time-harmonic solution to the equations of motion is represented as

2.20 $$\text{u}=\text{d}{\text{e}}^{-ik(\text{x}\cdot \text{n}-Vt)},$$

where *i*=(−1)^1/2^, *t* denotes time, **d** is the wave amplitude vector, **n** is the unit propagation vector and *k* is the wavenumber, which may be complex. Moreover, the vector **x** denotes the position vector, *ω*=*kV* is the angular frequency assumed to be always real, and *V* is the phase velocity. A substitution of equation (2.20) into the equations of motion (2.19) leads to the propagation condition

2.21 $$\left(\text{A}-\rho \omega^{2}\text{I}\right)\text{d}=\text{0},$$

where the *Cosserat acoustic tensor* **A**≡**A**(*k*,**n**) can be decomposed into a classical part ${\text{A}}^{\left(C\right)}$ and an additional couple-stress part ${\text{A}}^{\left(B\right)}$ as [12,20]

2.22 $$\text{A}=k^{2}{\text{A}}^{\left(C\right)}+k^{4}{\text{A}}^{\left(B\right)},$$

with

2.23 $${\text{A}}^{\left(C\right)}\equiv {\text{A}}^{\left(C\right)}(\text{n})=\left[\begin{array}{cc} c_{11}n_{1}^{2}+c_{66}n_{2}^{2} & (c_{12}+c_{66})n_{1}n_{2}\\ (c_{12}+c_{66})n_{1}n_{2} & c_{66}n_{1}^{2}+c_{22}n_{2}^{2} \end{array}\right]$$

and

2.24 $${\text{A}}^{\left(B\right)}\equiv {\text{A}}^{\left(B\right)}(\text{n})=\left(\eta_{1}n_{1}^{2}+\eta_{2}n_{2}^{2}\right)\left[\begin{array}{cc} n_{2}^{2} & -n_{1}n_{2}\\ -n_{1}n_{2} & n_{1}^{2} \end{array}\right],$$

where *n*_*q*_ (*q*=1,2) are the components of the propagation unit vector. Note that, since both ${\text{A}}^{\left(C\right)}$ and ${\text{A}}^{\left(B\right)}$ are symmetric, the acoustic tensor is also symmetric. It is remarked that the components of the acoustic tensor are *non-homogeneous* polynomials of fourth degree with respect to the wavenumber *k*. Hence, contrary to the classical elasticity case, the frequency and the phase velocity depend, in general, on the wavenumber, which implies that waves are dispersive in the context of the couple-stress theory. Note that in the context of micropolar (unconstrained Cosserat) theory the acoustic tensor and the conditions of (SE) were obtained in [21,22].

A necessary and sufficient condition for plane waves to propagate with positive speed and for all real wavenumbers *k* is that *the* *acoustic tensor is* *positive definite*, which is now defined as the (WP) condition

2.25 $$\text{p}\cdot \text{Ap}>0\Leftrightarrow k^{2}\left(\text{p}\cdot {\text{A}}^{\left(C\right)}\text{p}+k^{2}\text{p}\cdot {\text{A}}^{\left(B\right)}\text{p}\right)>0,$$

for every unit vector **n** and **p**. Thus, if the WP condition holds, the squared speeds (or equivalently the eigenvalues of the acoustic tensor) corresponding to each real acoustical axis are positive. For the plane-strain case considered here, two linearly independent plane waves always exist for a given direction of propagation **n** and real wavelength *k*. For small wavenumbers *k*→0 (low frequencies) equation (2.25) reveals that the classical part ${\text{A}}^{\left(C\right)}$ dominates the behaviour of the acoustic tensor, whereas for large wavenumbers k→∞ (high frequencies) the acoustic tensor is determined by its couple-stress part ${\text{A}}^{\left(B\right)}$. Therefore, taking into account that equation (2.25) must hold for all real non-zero wavenumbers, the (WP) condition in couple-stress elasticity is equivalent to the following pair of inequalities:

2.26 $$\text{p}\cdot {\text{A}}^{\left(C\right)}\text{p}\geq 0\;\text{and}\;\text{p}\cdot {\text{A}}^{\left(B\right)}\text{p}\geq 0,\;\forall \text{p}\neq \text{0},$$

augmented with the additional requirement that **p**⋅**Ap**≠0, so that both ‘=0’ cannot simultaneously apply in equation (2.26). In fact, the above conditions imply that, when ${\text{A}}^{\left(C\right)}$ and ${\text{A}}^{\left(B\right)}$ are *coaxial*, **p** cannot be an eigenvector corresponding to a *null* eigenvalue for both the classical and the couple-stress parts of the acoustic tensor.

An important property of the acoustic tensor is that its couple-stress part ${\text{A}}^{\left(B\right)}$ is singular and the propagation vector **n** is always an eigenvector of ${\text{A}}^{\left(B\right)}$ corresponding to a null eigenvalue

2.27 $$\det{\text{A}}^{\left(B\right)}=0\;\text{and}\;{\text{A}}^{\left(B\right)}\text{n}=\text{0},$$

so that the eigenvalues of ${\text{A}}^{\left(B\right)}$ are

2.28 $$\lambda_{1}(\text{n})\equiv 0\;\text{and}\;\lambda_{2}(\text{n})=\eta_{1}n_{1}^{2}+\eta_{2}n_{2}^{2}.$$

An immediate consequence of equation (2.27)_2_ is that, when **p** is *parallel* to the propagation vector **n**, equations (2.26) reduce to

2.29 $$\text{n}\cdot {\text{A}}^{\left(C\right)}\text{n}>0,$$

which is equivalent to the following inequalities for the Cauchy moduli:

2.30 $$c_{11}>0,\;c_{22}>0\;\text{and}\;-2c_{66}-\sqrt{c_{11}c_{22}}<c_{12}.$$

On the other hand, when the propagation vector is assumed to be aligned with the axes of orthotropy and by choosing **p**⋅**n**=0, inequalities (2.26) in conjunction with equation (2.28)_2_ imply that $c_{12}\leq \sqrt{c_{11}c_{22}},$ and {λ_2_≥0 and *c*_66_>0} or {λ_2_>0 and *c*_66_≥0}.

As a conclusion, the *necessary and sufficient* conditions for waves to propagate in all directions **n** and for all wavenumbers in an orthotropic constrained Cosserat material are

2.31 $$\text{(WP)}\Leftrightarrow \left\{ \begin{array}{l} c_{11}>0,\;c_{22}>0,\;-2c_{66}-\sqrt{c_{11}c_{22}}<c_{12}\leq \sqrt{c_{11}c_{22}}\\ \left\{ \begin{array}{l} \eta_{1}\geq 0,\;\eta_{2}\geq 0,\;\eta_{1}+\eta_{2}\neq 0\;\text{and}\;c_{66}>0\\ \text{or}\\ \eta_{1}>0,\;\eta_{2}>0\;\text{and}\;c_{66}\geq 0. \end{array}\right. \end{array}\right.$$

Note that, when $c_{12}=\sqrt{c_{11}c_{22}}$ or *c*_66_=0 with all the other strict inequalities satisfied in equation (2.31), (PD) and (SE) are lost simultaneously, but waves can still propagate. Therefore, (SE) is only a sufficient condition for WP in a constrained Cosserat material. This finding is in marked contrast to the classical elasticity case where the conditions of (SE) and (WP) are equivalent and provided by equations (2.17). It will be shown in §3 that the (WP) condition plays a major role for the derivation of the infinite-body Green's functions. In what follows it will always be assumed that the (WP) conditions hold for the constrained Cosserat material.

For the orthotropic material under consideration, pressure (*P*) and shear (*S*) waves exist when the propagation vector **n** is aligned parallel to the axes of the material orthotropy. In particular, for **n**=**e**_*α*_ (*α*=1,2), the wave velocities in directions 1 and 2 are given by

2.32 $$V_{P}^{\left(\alpha \right)}=\sqrt{\rho^{-1}c_{\alpha \alpha }}\;\text{and}\;V_{S}^{\left(\alpha \right)}=\sqrt{\rho^{-1}(c_{66}+\eta_{\alpha }\;k^{2})},\;\alpha =1,2,$$

where the index *α* is not summed. Note that in all other directions *mixed* type waves propagate with velocities that depend upon the wavenumber *k*.

Equation (2.32)_2_ reveals that for a couple-stress material with zero shear modulus (*c*_66_=0) the phase velocity of the shear wave becomes a linear function of the wavenumber, thus resembling the propagation of flexural harmonic waves in an Euler–Bernoulli beam [23]. On the other hand, when one of the Cosserat bending rigidities becomes zero, a shear wave travels without dispersion and with the classical velocity along the pertinent axis of orthotropy.

A final comment pertains to the special case in which $c_{12}=\sqrt{c_{11}c_{22}},$ where in a classical Cauchy material the velocity of a wave with characteristics **n**⋅**e**_1_=±[1+(*c*_11_/*c*_22_)^1/2^]^−1/2^ and **d**⋅**e**_1_=∓(*c*_11_/*c*_22_)^1/4^ becomes zero and, consequently, the (WP) condition is violated. On the contrary, when couple-stress effects are taken into account the (WP) condition is restored and waves can propagate in all directions.

### (c) Ellipticity

The structure of the differential operator (2.13) is now examined to define the condition of (E) relevant to the system of PDEs (2.10), the loss of which is connected to the emergence of various kinds of discontinuities. Assuming zero body forces and moments, the *symbol* ***l*** associated with the operator **L** is defined as [24]

2.33 $$\text{l}(\text{i}\text{k})=k^{2}{\text{A}}^{\left(C\right)}+k^{4}{\text{A}}^{\left(B\right)},$$

where **k**=*k***n** is an arbitrary real vector, of which **n** singles out its direction. The symbol is thus identified with the acoustic tensor, ***l***(i**k**)≡**A**(*k*,**n**), while its *principal part* ***l***^*P*^ is related to the principal operator **L**^*P*^, with $l^{P}\equiv k^{4}{\text{A}}^{\left(B\right)}.$ An immediate consequence of equation (2.27)_1_ is that

2.34 $$\det{\text{l}}^{P}=0,$$

showing that the principal part of the symbol is *degenerate*, so that the system of PDEs in couple-stress elasticity is not elliptic in the standard sense. A way to derive the (E) conditions in a general anisotropic constrained Cosserat material was given by Gourgiotis & Bigoni [12]. For a constrained orthotropic Cosserat material in the case of plane strain, the (E) condition assumes the following form:

2.35 $$(\text{E})\Leftrightarrow \text{n}\cdot {\text{A}}^{\left(C\right)}\text{n}\neq 0\;\text{and}\;\lambda_{2}(\text{n})\neq 0\;\forall \text{n}:|\text{n}|=1,$$

where λ_2_(**n**) is the eigenvalue of ${\text{A}}^{\left(B\right)}$ given in equation (2.28)_2_. The above conditions are equivalent to the following inequalities for the material moduli:

2.36 $$(\text{E})\Leftrightarrow c_{11}c_{22}\neq 0,\;c_{12}\neq -2c_{66}-\sqrt{c_{11}c_{22}}\;\text{and}\;\eta_{1}\eta_{2}\neq 0.$$

Therefore, it is apparent from equations (2.17) and (2.18) that (SE) of the elasticity tensors in a constrained Cosserat material implies (E) of the couple-stress equations. Note that the (E) conditions for a classical Cauchy orthotropic material are given by equations (2.36)_1_ and (2.36)_2_, augmented by the relations *c*_66_≠0 and $c_{12}\neq \sqrt{c_{11}c_{22}}$.

In what follows, unless otherwise stated, it will be assumed that *c*_22_>0 and *η*_2_>0. Under these circumstances, loss of (E) is attained when either *η*_1_=0 or *c*_11_=0. In both cases, the conditions of (E), (SE) and (PD) fail simultaneously. The special case where $c_{12}=-2c_{66}-\sqrt{c_{11}c_{22}}$ will not be considered in this study since (PD) is lost before (SE) (cf. equations (2.10)). It will be shown that, when *η*_1_=0, loss of (E) triggers new phenomena such as folding and faulting that cannot be described by the classical theory. On the other hand, when *c*_11_=0, dilation/compaction bands will be shown to emerge qualitatively similar to those evidenced in a classical continuum.

## 3. Green's functions for a concentrated force and a concentrated moment

The field equations governing plane-strain deformations in the case of an orthotropic Cosserat material admit two infinite-body Green's functions: one for an in-plane concentrated force **P**=(*P*_1_,*P*_2_) *δ*(**x**), and one for an out-of-plane concentrated moment *Mδ*(**x**). The components of the generalized force vector **F** in the equilibrium equations (2.12) can then be written as

3.1 $$F_{1}=P_{1}\delta (x_{1})\delta (x_{2})+\frac{M}{2}\delta (x_{1})\delta^{\prime }(x_{2})\;\text{and}\;F_{2}=P_{2}\delta (x_{1})\delta (x_{2})-\frac{M}{2}\;\delta^{\prime }(x_{1})\delta (x_{2}),$$

where *δ*() denotes the Dirac delta distribution and the prime implies differentiation with respect to the relevant variable.

An exact solution to equation (2.12) is obtained by employing the double exponential Fourier transform. The direct and inverse double Fourier transforms are defined as

3.2 $$\tilde{f}(\text{k})=\int_{-\infty }^{+\infty }\int_{-\infty }^{+\infty }f(\text{x})\;e^{i\text{k}\cdot \text{x}}\;dx_{1}\;dx_{2}\;\text{and}\;f(\text{x})=\frac{1}{4\pi^{2}}\int_{-\infty }^{+\infty }\int_{-\infty }^{+\infty }\tilde{f}(\text{k})\;e^{-i\text{k}\cdot \text{x}}\;dk_{1}\;dk_{2},$$

where **x**=(*x*_1_,*x*_2_) and **k**=(*k*_1_,*k*_2_) is the Fourier vector with **k**=*k***n**.

### (a) Concentrated force

In the case of a concentrated force, the Green's function is derived by applying the direct double Fourier transform (3.2)_1_ to the field equations (2.12) with *M*=0, yielding the following solution for the displacement field:

3.3 $$u_{q}(\text{x})=\frac{P_{p}}{4\pi^{2}}\int_{-\infty }^{\infty }\int_{-\infty }^{\infty }\frac{C_{pq}(\text{k})}{D(\text{k})}\;e^{-i\text{k}\cdot \text{x}}\;dk_{1}\;dk_{2},$$

where *C*_*pm*_(**k**)=Cof[*A*_*pm*_(*k*,**n**)] is the cofactor of the acoustic tensor with components

3.4 $$\begin{array}{rl} C_{11} & =c_{66}k_{1}^{2}+c_{22}k_{2}^{2}+k_{1}^{2}\left(\eta_{1}k_{1}^{2}+\eta_{2}k_{2}^{2}\right),\\ C_{12} & =C_{21}=-k_{1}k_{2}\left(c_{12}+c_{66}-\left(\eta_{1}k_{1}^{2}+\eta_{2}k_{2}^{2}\right)\right)\\ \text{and}\;C_{22} & =c_{11}k_{1}^{2}+c_{66}k_{2}^{2}+k_{2}^{2}\left(\eta_{1}k_{1}^{2}+\eta_{2}k_{2}^{2}\right), \end{array}\}$$

and *D*(**k**) is the characteristic polynomial identified with the determinant of the acoustic tensor

3.5 $$D(\text{k})\equiv \det\text{A}=C_{11}C_{22}-C_{12}^{2}.$$

Note that by setting *η*_1_=*η*_2_=0 the solution (3.3) degenerates to the classical elasticity solution given in appendix A.

For a fixed value of the transformed variable *k*_1_ ($k_{1}\in R$), *D*(**k**) is a *sextic non-homogeneous* polynomial of the variable *k*_2_, which has *no* real roots when the (WP) condition holds (i.e. when the acoustic tensor is positive definite). In particular, for each fixed value of *k*_1_, there are three roots $k_{2}^{\left(m\right)}\equiv k_{2}^{\left(m\right)}(k_{1})$ satisfying

3.6 $$D\left(k_{1},k_{2}^{\left(m\right)}\right)=0\;\text{and}\;\text{Im}\left[k_{2}^{\left(m\right)}\right]<0\;(m=1,2,3),$$

so that the characteristic polynomial can be written as

3.7 $$D(\text{k})=c_{22}\eta_{2}\prod_{m=1}^{3}\left(k_{2}-k_{2}^{\left(m\right)}\right)\left(k_{2}-{\bar{k}}_{2}^{\left(m\right)}\right),$$

where ${\bar{k}}_{2}^{\left(m\right)}$ are the complex conjugates of $k_{2}^{\left(m\right)}$. For an orthotropic Cosserat material the roots of the characteristic polynomial are always distinct (single) and can be analytically evaluated through Cardano's formula.

Applying the residue theorem in conjunction with Jordan's lemma, the integration with respect to *k*_2_ in equation (3.3) yields a summation of residues of poles at $k_{2}=k_{2}^{\left(m\right)}$ when *x*_2_>0 or at $k_{2}={\bar{k}}_{2}^{\left(m\right)}$ when *x*_2_<0. In particular, for *x*_2_>0, the original integration path running along the real axis in the *k*_2_-plane is replaced by a closed contour taken in the lower *k*_2_-plane so that the integrand is decaying as $|k_{2}|\to \infty .$ In this case, the following relation is obtained:

3.8 $$\int_{-\infty }^{\infty }\frac{C_{pq}(\text{k})\;{\text{e}}^{-\text{i}k_{2}x_{2}}}{D(\text{k})}\;\text{d}k_{2}=-2\pi \text{i}Q_{pq}(k_{1},x_{2}),$$

with

3.9 $$Q_{pq}(k_{1},x_{2})=\sum_{m=1}^{3}{\left[\frac{C_{pq}(\text{k})\;{\text{e}}^{-\text{i}k_{2}x_{2}}}{\partial_{k_{2}}D(\text{k})}\right]}_{k_{2}=k_{2}^{\left(m\right)}},$$

where ∂_*k*_2__≡∂()/∂*k*_2_.

Bearing in mind that the components of *Q*_*pq*_ are even functions of *k*_1_ when *p*=*q*, and odd functions when *p*≠*q*, the displacement components due to a concentrated force can be written as

3.10 $$\begin{array}{rl} u_{1}(\text{x}) & =\frac{P_{1}}{\pi }\;\text{FP}\int_{0}^{\infty }{\hat{Q}}_{11}(k_{1},x_{2})\cos(k_{1},x_{1})\;dk_{1}+\frac{P_{2}}{\pi }\int_{0}^{\infty }{\hat{Q}}_{12}(k_{1},x_{2})\sin(k_{1},x_{1})\;dk_{1}\\ \text{and}\;u_{2}(\text{x}) & =\frac{P_{1}}{\pi }\int_{0}^{\infty }{\hat{Q}}_{12}(k_{1},x_{2})\sin(k_{1},x_{1})\;dk_{1}+\frac{P_{2}}{\pi }\;\text{FP}\int_{0}^{\infty }{\hat{Q}}_{22}(k_{1},x_{2})\cos(k_{1},x_{1})\;dk_{1}, \end{array}\}$$

where

3.11 $${\hat{Q}}_{pq}=\left\{ \begin{array}{l} \text{Im}[Q_{pq}],\;p=q\\ -\text{Re}[Q_{pq}],\;p\neq q. \end{array}\right.$$

The integrals in equation (3.10) can be numerically evaluated by taking into account the oscillatory character of their integrands. Accordingly, the strain and stress components are computed by direct substitution of the displacement solution into equations (2.2) and (2.7)–(2.9). Note that the Fourier cosine integrals in equation (3.10) must be evaluated in the Hadamard finite part (FP) sense due to the behaviour of their integrands as *k*_1_→0 [13]. In particular, the following asymptotic behaviour can be demonstrated:

3.12 $$\lim_{k_{1}\to 0}\;{\hat{Q}}_{pq}(k_{1},x_{2})=O\left(k_{1}^{-1}\right)\;\text{and}\;\lim_{k_{1}\to \infty }{\hat{Q}}_{pq}(k_{1},x_{2})=O\left(k_{1}^{-1}e^{-a\;x_{2}}\right),\;\text{for}\;p=q,$$

with a=minm=1,2,3{limk1→∞|Im[k2(m)(k1)]|}. Therefore, employing the Abel–Tauber theorem [25], it can be readily shown that the displacement component parallel to the direction of the applied load is *logarithmically* divergent at the origin (where the force is applied) and at infinity, as in the classical theory. The latter observation implies that the displacement field can be evaluated within a rigid body motion which is inherently indeterminate [26]. This indeterminacy is characteristic also of two-dimensional concentrated load problems in the context of couple-stress elasticity [13,27]. Finally, it is remarked that the strain and stress components exhibit a *Cauchy*-type singularity at the point of application of the concentrated load.

### (b) Concentrated moment

For an applied concentrated moment (**P**=0), the solution for the displacement field assumes the following form:

3.13 $$u_{q}(\text{x})=\frac{M}{4\pi^{2}}\int_{-\infty }^{\infty }\int_{-\infty }^{\infty }\frac{\text{i}\;e_{3np}k_{n}C_{pq}(\text{k})}{2D(\text{k})}\;{\text{e}}^{-\text{i}\text{k}\cdot \text{x}}\;\text{d}k_{1}\;\text{d}k_{2},$$

where *e*_*qnp*_ is the Levi–Civita alternate tensor. A procedure analogous to that followed before to obtain Green's function for applied concentrated load can be employed for the derivation of Green's function for applied moment. In this case, the displacement field can be written as

3.14 $$\begin{array}{rl} u_{1}(\text{x}) & =\frac{M}{\pi }\int_{0}^{\infty }\text{Im}[N_{1}(k_{1},x_{2})]\cos(k_{1}x_{1})\;dk_{1}\\ \text{and}\;u_{2}(\text{x}) & =-\frac{M}{\pi }\int_{0}^{\infty }\text{Re}[N_{2}(k_{1},x_{2})]\sin(k_{1}x_{1})\;dk_{1}, \end{array}\}$$

with

3.15 $$N_{q}(k_{1},x_{2})=i\sum_{m=1}^{3}{\left[\frac{e_{3np}k_{n}C_{pq}(\text{k})\;{\text{e}}^{-\text{i}k_{2}x_{2}}}{2\partial_{k_{2}}D(\text{k})}\right]}_{k_{2}=k_{2}^{\left(m\right)}}.$$

The functions *N*_*q*_(*k*_1_,*x*_2_) (*q*=1,2) are bounded in $k_{1}\in [0,\infty )$, which implies that the integrals in equations (3.14) are convergent and can be directly evaluated numerically taking into account their oscillatory character. Note that, when *x*_2_<0, the summation in equation (3.15) should be performed at $k_{2}={\bar{k}}_{2}^{\left(m\right)}$ (i.e. the poles at the upper-half of the *k*_2_-plane). In contrast to the case of the concentrated load, the displacement components for an applied concentrated moment in an infinite orthotropic couple-stress medium are *bounded* both at the origin and at infinity. An analogous result was found by Weitsman [28] in the case of an isotropic couple-stress material.

A case that merits special attention is when the material has a null bending modulus in the *x*_1_-direction, *η*_1_=0, so that (E) is lost (equation (2.36)), but the (WP) condition (2.31) still holds. The characteristic polynomial *D*(**k**) in this case has no real roots, and Green's functions (3.10) and (3.14) still apply. This finding is in marked contrast with the classical elasticity situation where loss of (E) implies also loss of the (WP) condition (i.e. the classical acoustic tensor is no longer positive definite) and, thus, Green's function can only be defined in the sense of distributions (appendix A).

In the following, the above derived Green's functions will be used as perturbing agents in several cases. Note that the Cosserat orthotropic material under plane-strain conditions is characterized effectively by four dimensionless parameters, namely *α*=*c*_11_/*c*_22_, *δ*=*c*_12_/*c*_22_, *ε*=*c*_66_/*c*_22_ and *β*=*η*_1_/*η*_2_, where it is recalled that *c*_22_>0 and *η*_2_>0. The ratio *β* measures the degree of Cosserat anisotropy, so that for an extreme Cosserat material the ratio *β* tends to zero or to infinity.

### (c) The Cosserat length

The typical problem arising when Cosserat or other generalized continua are analysed is how to relate the internal constitutive length to the microstructure present in the real material under modelling. Here, several approaches are possible [7,8,29–31], but, as applications to geophysics will be discussed later, Biot's approach will be followed. In this context, two independent characteristic lengths $\tilde{\ell }$ and ℓ can be introduced in the constitutive relations through: $\eta_{1}=c_{11}{\tilde{\ell }}^{2}$ and *η*_2_=*c*_22_ℓ^2^. For a layered rock material where stiff strata of thickness *h* are alternated with compliant strata [7] (his eqn 7.11, where *b*=2*η*_1_; see also [8]) provides the following relation for the internal length: $\tilde{\ell }=h/\sqrt{6}$. A value representative of the layers reported in figure 1*a*,*b* is *h*≈25 cm, so that $\tilde{\ell }\approx 10\;\text{cm}$.

## 4. Folding of an elastic continuum and the formation of a chevron structure

Folding in a constrained Cosserat solid *near* the loss of (E) is revealed through a perturbation of the material by a concentrated force. The perturbation technique was introduced by Bigoni & Capuani [32] for elastic prestressed materials and is thoroughly discussed by Bigoni [15]. During folding, certain components of the displacement gradient suffer a *finite* jump across a discontinuity surface, while the displacement components become locally piecewise smooth. In the case of an orthotropic couple-stress material under plane-strain conditions, folding occurs when the ratio *β*=*η*_1_/*η*_2_ tends to zero or to infinity, so that loss of (E) (of the Cosserat part of the constitutive tensors) is attained (equation (2.36)_3_).

**Figure 3.**
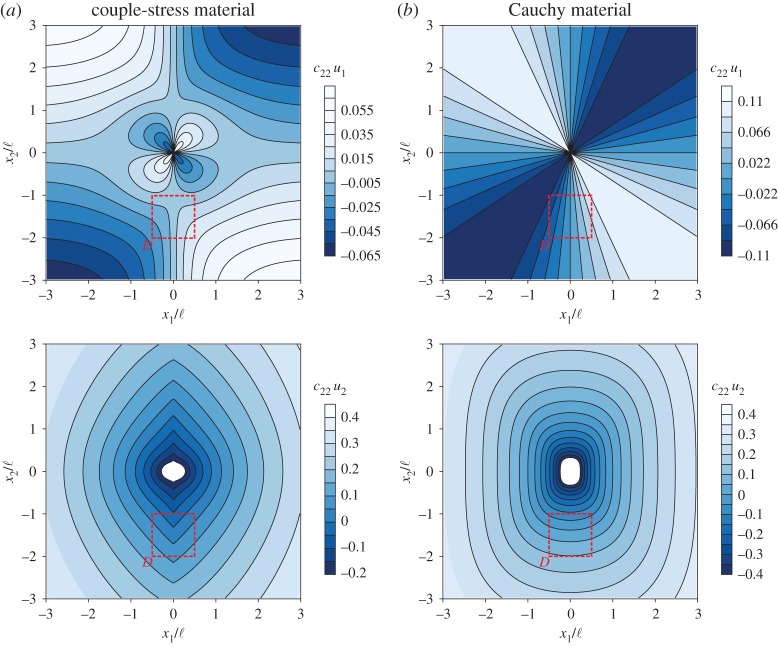
Dimensionless level sets of the displacement components *c*_22_*u*_1_ and *c*_22_*u*_2_ due to a concentrated unit force acting at the origin of the axes and aligned with the *x*_2_-axis of orthotropy. (*a*) Couple-stress material at the failure of (E) (*α*=0.5, *ε*=0.5, *δ*=0.2, *β*=0) where folding is clearly visible. (*b*) The underlying classical Cauchy material (*α*=0.5, *ε*=0.5, *δ*=0.2) far from (E) loss where localization is not observed. (Online version in colour.)

Figure 3 depicts the level sets of the dimensionless displacement components *c*_22_*u*_1_ and *c*_22_*u*_2_ produced by a concentrated unit force **P**=(0,−1), aligned with the *x*_2_-axis of orthotropy. Note that in all the following figures the coordinates (*x*_1_,*x*_2_) are normalized with the characteristic length ℓ of the couple-stress material. More specifically, figure 3*a* shows that, in a couple-stress material at the failure of (E) (*α*=0.5, *ε*=0.5, *δ*=0.2 and *β*=0), the normalized vertical displacement *c*_22_*u*_2_ becomes piecewise smooth (so that a vertex is displayed) across the discontinuity line *x*_1_=0. At the origin of the axes, the vertical displacement remains logarithmically unbounded as in the classical theory. It is worth noting that, for the couple-stress material under investigation, both (E) and (PD) are lost simultaneously when *η*_1_=0, but the (WP) condition still holds, so that Green's function (3.3) is well defined. In fact, it is rather remarkable that at the failure of (E) the displacement components remain bounded (apart from the origin) even on the line of discontinuity (*x*_1_=0). On the other hand, figure 3*b* depicts the response of the underlying classical Cauchy material without Cosserat effects (*α*=0.5, *ε*=0.5, *δ*=0.2), which, according to equation (2.10)_1_, is PD (far from (E) loss) and, thus, no localization is observed.

For a classical Cauchy material, the counterpart of the condition *β*=0 is that (E) is lost when the shear modulus becomes zero (*ε*=0). In this case, the component of the displacement aligned with the direction of the concentrated force exhibits a Dirac-type localization (equation (9.6)) along the *whole* discontinuity line (*x*_1_=0). This is in sharp contrast with the situation where *β*=0 in a couple-stress material where (E) is lost, but the displacement remains bounded and exhibits a *cusp* along the discontinuity line (with the exception of the point of application of the concentrated force).

**Figure 4.**
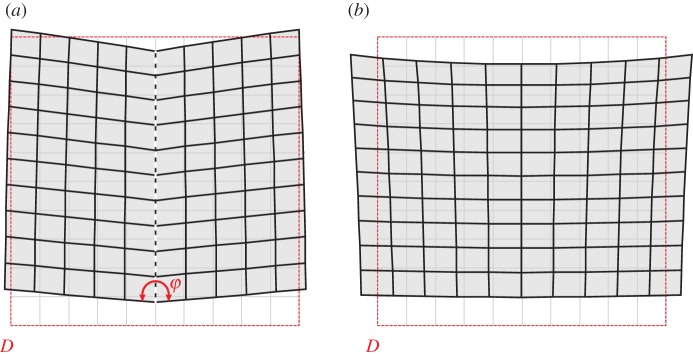
The actual deformed shape of a rectangular region *D* referred to the undeformed configuration. (*a*) Single localized in-plane folding is formed in a couple-stress material at the failure of (E) (*α*=0.5, *ε*=0.5, *δ*=0.2, *β*=0). The folding angle *φ* varies with the distance from the point of application of the concentrated force. (*b*) The underlying classical Cauchy material (*α*=0.5, *ε*=0.5, *δ*=0.2) shows a diffused, mild, bending. (Online version in colour.)

The formation of folding in the couple-stress material is more clearly depicted in figure 4, where the actual deformed shape of a rectangular region referred to the undeformed configuration *D*≡{(*x*_1_,*x*_2_): |*x*_1_|≤0.5ℓ,−ℓ≤*x*_2_≤−2ℓ} (highlighted with a red rectangle in figure 3) is shown for both the couple-stress and the underlying classical materials. It is observed that the couple-stress material (figure 4*a*) folds along the discontinuity line *x*_1_=0 (white/black dashed line) forming a single (chevron-type) in-plane crease. The lateral sides of the region *D* remain almost straight and an extremely localized bending (curvature tending to infinity) occurs on the line where the material folds. This situation closely resembles the folding formation in layered rocks (figure 1) and is in marked contrast with the behaviour of the underlying classical Cauchy material where all sides of the region *D* undergo a *small-curvature* diffused bending (figure 4*b*).

The conditions under which the formation of a discontinuity line becomes possible at (E) loss are now examined for an orthotropic couple-stress material under plane-strain conditions (the general conditions for a three-dimensional anisotropic body were obtained by Gourgiotis & Bigoni [12]). From equilibrium considerations and imposing continuity of displacements across the surface defined by the unit normal **n**=(1,0), the following relations are derived:

4.1 $$[[u_{1}]]=0,\;[[u_{2}]]=0$$

and

4.2 $$[[\sigma_{11}]]=0,\;[[\sigma_{12}]]=0,\;[[m_{13}]]=0,$$

where [[ ]] denotes the jump of the enclosed quantity across the discontinuity surface. In addition, employing Hadamard's lemma [33], it can be shown that: $[[\partial_{2}^{p}u_{q}]]=\partial_{2}^{p}[[u_{q}]]=0$ with $p\in Z_{+}.$

Using the kinematical conditions (2.2) in conjunction with the constitutive equations (2.7)–(2.9), the equations for the continuity of tractions in (4.2) assume, respectively, the form

4.3 $$c_{11}g_{1}^{\left(1\right)}=0,\;c_{66}g_{2}^{\left(1\right)}-\eta_{2}\frac{{\text{d}}^{2}g_{2}^{\left(1\right)}}{\text{d}x_{2}^{2}}-\eta_{1}g_{2}^{\left(3\right)}+\eta_{1}\frac{\text{d}g_{1}^{\left(2\right)}}{\text{d}x_{2}}=0,\;\eta_{1}\left(g_{2}^{\left(2\right)}-\frac{\text{d}g_{1}^{\left(1\right)}}{\text{d}x_{2}}\right)=0,$$

where **g**^(*p*)^≡**g**^(*p*)^(*x*_2_) is the discontinuity vector of the *p*th-order whose components are the jumps in the normal derivatives of the displacements: $g_{q}^{\left(p\right)}\equiv [[\partial_{1}^{p}u_{q}]].$ The algebraic-differential system (4.3) is underdetermined since it consists of three equations with five unknowns, namely the components of the discontinuity vectors. The system becomes determinate when (E) associated with the Cosserat moduli fails. In particular, when (E) is lost for *η*_1_=0 (but *η*_2_>0), the system (4.3) reduces to

4.4 $$c_{11}g_{1}^{\left(1\right)}=0\;\text{and}\;\eta_{2}\frac{{\text{d}}^{2}g_{2}^{\left(1\right)}}{\text{d}x_{2}^{2}}-c_{66}g_{2}^{\left(1\right)}=0.$$

Note that the case where failure of (E) occurs when *η*_2_=0 (*η*_1_>0) can be treated in a strictly analogous manner.

Assuming that the (WP) condition (2.31) holds, equations (4.4) yield the solution

4.5 $$g_{1}^{\left(1\right)}(x_{2})=0\;\text{and}\;g_{2}^{\left(1\right)}(x_{2})=C{\text{e}}^{-|x_{2}|\sqrt{c_{66}/\eta_{2}}},$$

where *C* is a non-zero constant. The above result shows that, when (E) is lost but the (WP) condition still holds, a non-zero discontinuity vector **g**^(1)^ becomes possible with an exponentially decaying amplitude along the discontinuity line. Accordingly, the folding angle, $\varphi (x_{2})=\pi -g_{2}^{\left(1\right)}(x_{2})$ (figure 4*a*), increases with increasing distance from the origin and tends to the value *π* (corresponding to the absence of folding) as $|x_{2}|\to \infty$.

It is worth noting that the localization conditions in the constrained Cosserat theory are of algebraic-differential type [12], whereas in the classical theory the corresponding conditions degenerate to a purely algebraic equation of the form ${\text{A}}^{\left(C\right)}{\text{g}}^{\left(1\right)}=\text{0}$ [15]. The differential nature of the localization condition is general and pertains also to all generalized continuum theories of gradient type, for instance strain gradient elasticity.

**Figure 5.**
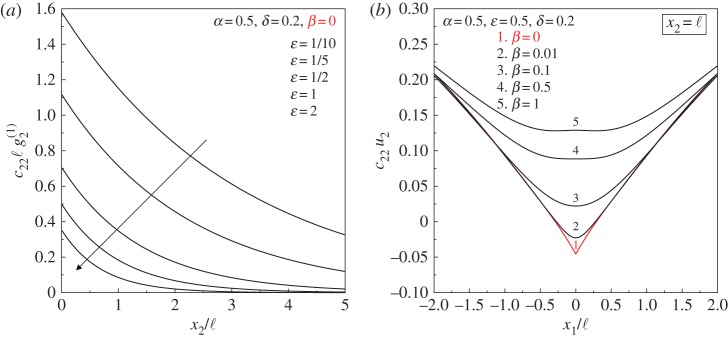
(*a*) Exponential decay of the dimensionless jump $c_{22}\ell g_{2}^{\left(1\right)}$ along the discontinuity line (*x*_1_=0) for various values of the ratio *ε* in a constrained Cosserat material. (*b*) Progressive formation of folding due to the action of a unit concentrated force as the ratio of the bending moduli *β* tends to zero and failure of (E) is approached. The dimensionless vertical displacement *c*_22_*u*_2_ is plotted at the level *x*_2_=ℓ. (Online version in colour.)

Figure 5*a* shows that the jump in the normal derivative of the vertical displacement, $g_{2}^{\left(1\right)}=[[\partial_{1}u_{2}]],$ is finite and decays exponentially along the discontinuity line, according to equation (4.5). The magnitude and the decay rate of the jump depend strongly upon the ratio *ε*. In fact, as *ε* decreases, the folding angle *φ* becomes more acute while the jump decays at a slower rate, which, in turn, implies that folding *channels* through the material. Note that when *β*=0 and *ε*→0, in addition to loss of (E), failure of the (WP) condition is also approached.

A final comment pertains to the fact that, although folding emerges in a Cosserat elastic material at the limit of (E) loss, rapid variations of the normal gradient are already visible near this limit, when (E) is still preserved.^4^ This situation is illustrated in figure 5*b*, where the progressive formation of folding is shown as the ratio *β* tends to zero. In particular, for *β*=0.01 (curve 2), a localized bending of high curvature is observed across the line *x*_1_=0. The fact that such a behaviour is clearly visible in the proximity (but still inside) of the border of the elliptic domain (and even when the strain energy function is still strictly positive) means that extreme materials such as those analysed in the present article *can be realized in practice* and employed to explore, as yet, unattained mechanical behaviour.

## 5. Faulting of an elastic continuum

Another new phenomenon evidenced in the context of the couple-stress theory is faulting. During faulting the displacement exhibits a *finite* localized jump across a discontinuity surface. This phenomenon emerges from the application of a concentrated *moment* to an extreme couple-stress material at the failure of (E) of the Cosserat part of the constitutive tensors.

**Figure 6.**
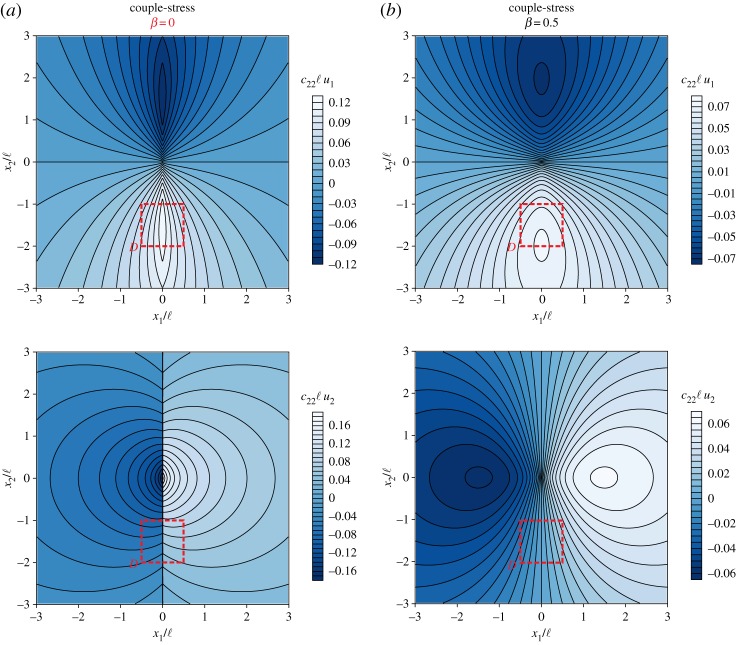
Dimensionless level sets of the displacement components *c*_22_ℓ*u*_1_ and *c*_22_ℓ*u*_2_ due to a concentrated unit moment acting at the origin of the axes. (*a*) A couple-stress material at the failure of (E) (*α*=0.5, *ε*=0.5, *δ*=0.2, *β*=0) evidences faulting. The vertical displacement becomes discontinuous along the line *x*_1_=0. (*b*) A couple-stress material far from (E) loss (*α*=0.5, *ε*=0.5, *δ*=0.2, *β*=0.5). The displacement field remains continuous and no localization is observed. (Online version in colour.)

Figure 6 depicts the level sets of the dimensionless displacement components *c*_22_ℓ*u*_1_ and *c*_22_ℓ*u*_2_ as produced by a concentrated out-of-plane unit moment (*M*=1). In figure 6*a*, it is shown that in a couple-stress material at the failure of (E) (*α*=0.5, *ε*=0.5, *δ*=0.2 and *β*=0) the vertical displacement *c*_22_ℓ*u*_2_ becomes discontinuous across the line *x*_1_=0. In figure 6*b*, the response of a positive definite couple-stress material (*α*=0.5, *ε*=0.5, *δ*=0.2 and *β*=0.5) shows that the displacement field is continuous and, thus, no localization is observed.

**Figure 7.**
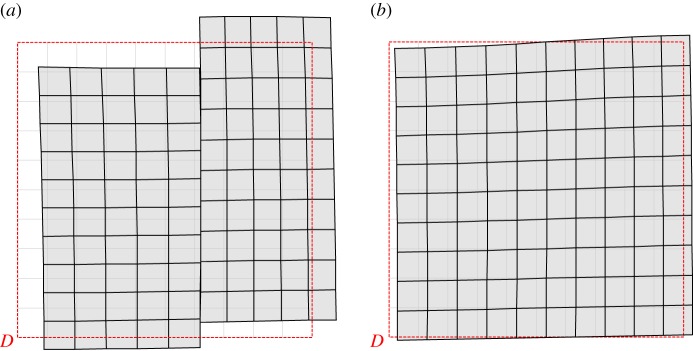
The actual deformed shape of a rectangle region *D* referred to the undeformed configuration due to a concentrated unit moment acting at the origin of the axes. (*a*) Faulting is formed in the material at the failure of (E) (*α*=0.5, *ε*=0.5, *δ*=0.2, *β*=0). (*b*) No localization is observed in the positive definite material (*α*=0.5, *ε*=0.5, *δ*=0.2, *β*=0.5). (Online version in colour.)

The formation of faulting is illustrated in figure 7, where the actual deformed shape of the rectangular region *D*≡{(*x*_1_,*x*_2_): |*x*_1_|≤0.5ℓ,−ℓ≤*x*_2_≤−2ℓ} referred to the undeformed configuration (red rectangle in figure 6) is shown for both the extreme (*β*=0) and non-extreme (*β*=0.5) couple-stress materials. It can be observed that at the failure of (E) (figure 7*a*) an in-plane slip discontinuity of *finite* width is formed. The magnitude of the jump in the displacement vector decreases with increasing distance from the point of application of the concentrated moment.

**Figure 8.**
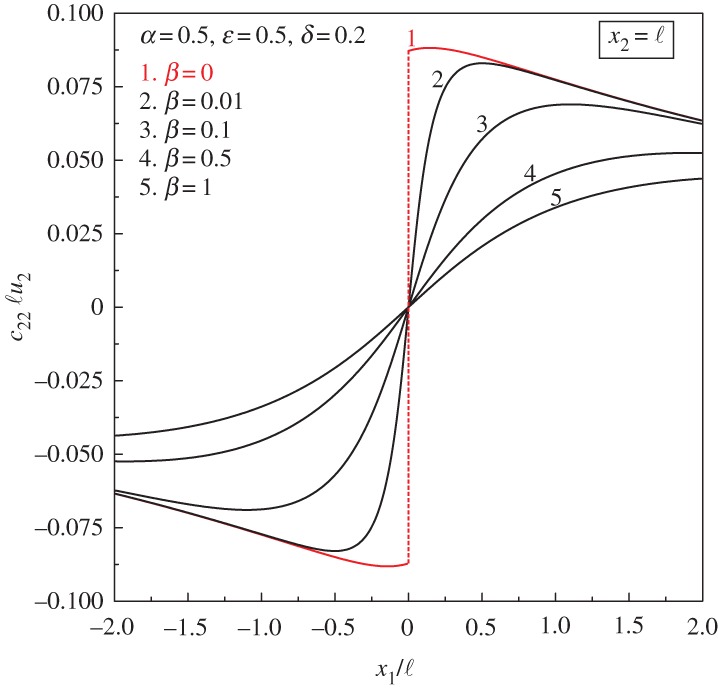
Progressive formation of faulting due to the action of a concentrated unit moment as the ratio of the bending moduli *β* tends to zero and failure of (E) is approached. The dimensionless vertical displacement *c*_22_ℓ*u*_2_ is plotted at the level *x*_2_=ℓ. (Online version in colour.)

Finally, figure 8 shows the progressive formation of faulting as the ratio *β* tends to zero and loss of (E) is approached. It is worth noting that, although faulting occurs at the failure of (E) (*β*=0), steep variations in the vertical displacement *c*_22_ℓ*u*_2_ are clearly visible near this border too.

## 6. Stress channelling

For classical Cauchy elastic materials with extreme orthotropic properties, the stress produced by a concentrated load has a slow diffusion, so that the solution becomes highly localized and strongly directional. In fact, in the limit when the stiffness ratio between different material directions tends to zero, the equilibrium equations reach the elliptic boundary and the stress percolates through *null-thickness* deformation bands. This phenomenon is called *stress channelling* (as theoretically analysed by Everstine & Pipkin [34] and experimentally proven by Bigoni & Noselli [35,36]) and has also been evidenced in constrained Cosserat elasticity under antiplane shearing [13].

**Figure 9.**
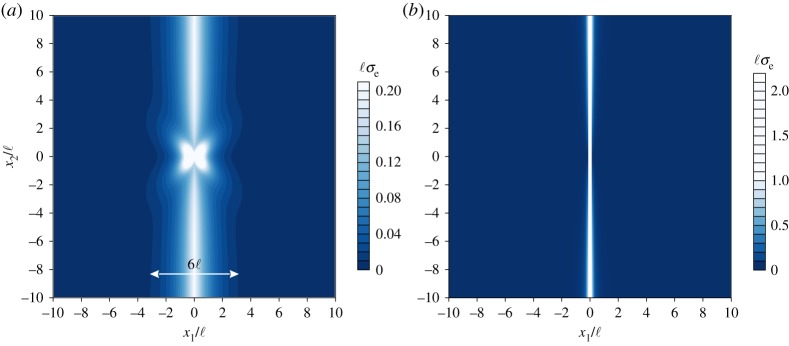
Dimensionless level sets of the von Mises stress ℓ*σ*_*e*_ due to a concentrated unit force. Approaching the limit of (E) loss, the stress channels into: (*a*) a band of *finite*thickness (of width 6ℓ) for a couple-stress material (*α*=0.5, *ε*=10^−9^, *δ*=0.2, *β*=10^−9^) and (*b*) a band of *null* thickness for a classical Cauchy material (*α*=0.5, *ε*=10^−9^, *δ*=0.2). (Online version in colour.)

In figure 9, the map of the dimensionless effective von Mises stress ℓ*σ*_*e*_ ($\sigma_{e}=\sqrt{3/2}∥\text{dev}\sigma ∥$) produced by a concentrated unit force **P**=(−1,0) which acts at the origin of the axes is reported for a constrained Cosserat material (*α*=0.5, *ε*=10^−9^, *δ*=0.2, *β*=10^−9^) and for the underlying Cauchy material (*α*=0.5, *ε*=10^−9^, *δ*=0.2), both being near to failure of (E). For the Cosserat material (figure 9*a*), it is observed that the effective stress localizes in a band of *finite* thickness as *β*→0 and channels through the material. The width of the localization band is approximately 6ℓ; outside this band the stress quickly decreases to zero. It is worth noting that, even at the failure of (E), the stress components remain bounded along the discontinuity line (*x*_1_=0), except at the point of application of the concentrated force. It is further remarked that the stress components converge to the same (finite) limit in the two cases *ε*→0, *β*=0 and *ε*=0, *β*→0. On the other hand, an extreme localization is observed for the classical Cauchy material (figure 9*b*), yielding an effective stress concentrated along a band of *null thickness*. In fact, in the limit of vanishing shear modulus (*ε*=0) E is lost in the underlying classical material (see §2.3) and the von Mises stress exhibits a Dirac delta discontinuity across the line *x*_1_=0 (appendix A).

**Figure 10.**
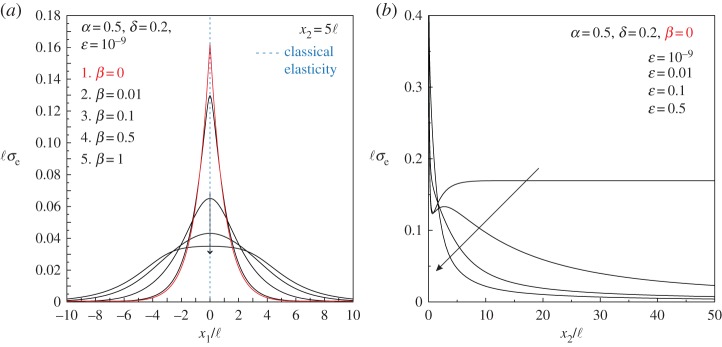
Profiles of the dimensionless von Mises stress ℓ*σ*_*e*_ due to a concentrated unit force. (*a*) The progressive localization of the von Mises stress plotted at the level *x*_2_=5ℓ, as the ratio *β* tends to zero and the material approaches failure of (E). (*b*) The variation of the von Mises stress along the discontinuity line (*x*_1_=0) for a couple-stress material at (E) loss (*β*=0) shows that stress channelling occurs as the shear modulus tends to zero *ε*. (Online version in colour.)

The progressive stress localization as the couple-stress material approaches (E) loss is more clearly shown in figure 10*a*, where profiles of the dimensionless von Mises stress ℓ*σ*_*e*_ are shown at the level *x*_2_=5ℓ for selected values of the ratio *β*. The stress localizes into bands of *finite width* (around the discontinuity line) which depends on the ratio *β*. At the failure of (E) (*β*=0), the von Mises stress becomes piecewise smooth, displaying a vertex along the line *x*_1_=0, but its value remains *finite*. The equivalent von Mises stress for the underlying classical Cauchy material exhibits a Dirac-type localization (dashed blue line). In figure 10*b*, the variation of the von Mises stress *along* the discontinuity line is reported for a couple-stress material at the failure of (E) (*β*=0) and for different values of the ratio *ε*. It is observed that the stress channels through the material *without* attenuation as the shear modulus tends to zero, so that the (WP) condition is also close to being violated.

In conclusion, it can be stated that stress localization is attributed to loss of (E) driven by Cosserat elastic moduli (controlled by the ratio *β*), whereas stress channelling through the material occurs when also the (WP) condition is close to failure (controlled by the ratios *β* and *ε*).

## 7. Dilation/compaction bands

A mode of localization different from folding and faulting also exists for couple-stress materials at (E) loss, namely the formation of dilation/compaction bands. These bands are localized planar zones formed normal to the highest tensile/compressive stress and are similar to the usual localization bands occurring in the non-polar case. The localization condition for dilation/compaction bands assumes the form **g**^(1)^×**n**=**0** [15]. Unlike the cases of folding and faulting, this type of localization occurs due to failure of (E) associated with the Cauchy moduli. In particular, for an orthotropic material dilation/compaction bands emerge when the ratio *α*=*c*_11_/*c*_22_ tends to zero or to infinity. In this case, the (WP) and (E) conditions are lost simultaneously (equations (2.31) and (2.36)_1_) for both the couple-stress and the underlying Cauchy materials.

**Figure 11.**
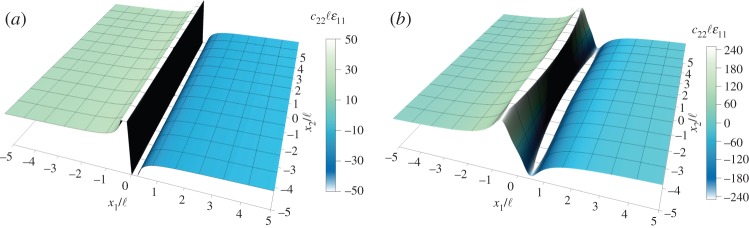
Normalized strain *c*_22_ℓ*ε*_11_ produced by a concentrated unit force acting at the origin of axes in the *x*_1_-direction. Similar compaction and dilation bands form near (E) loss (*a*) in the couple-stress material (*α*=10^−4^, *ε*=0.5, *δ*=0, *β*=0.5) and (*b*) in the underlying classical Cauchy material (*α*=10^−4^, *ε*=0.5, *δ*=0). (Online version in colour.)

The couple-stress material is perturbed by a concentered unit force **P**=(1,0), acting in the *x*_1_-direction, and is assumed to be *near* the elliptic border (*α*=10^−4^, *ε*=0.5, *δ*=0 and *β*=0.5). Figure 11 depicts the localization of the dimensionless strain *c*_22_ℓ*ε*_11_ in narrow compaction (*x*_1_>0) and dilation (*x*_1_<0) bands, normal to the direction of the applied unit force. The response of the couple-stress material (figure 11*a*) and the underlying classical material (figure 11*b*) is qualitatively similar when *α*→0. This is attributed to the fact that in this case the couple-stress effects cannot restore (E) (equation (2.36)_1_).

## 8. An application to geophysics: chevron folding

Chevron folding occurs in layered rocks in which stiff strata alternate with compliant ones. In such cases, the orthotropy of the material plays a major role. This was pointed out by Biot [37,38] (see also [39–41]), who also showed that a layered material generates, in a homogenized sense, Cosserat effects. Chevron folding is a complex *evolutionary* phenomenon, involving time effects, buckling, and perhaps also chemical processes (a number of models have been developed to explain this phenomenon, see, for example, [1,42,43] and references therein). Although it is impossible to describe such a complexity in the constitutive framework employed in this article, it is shown below how chevron folding can emerge as related to a progressive change in the material properties, so that the border of Cosserat (E) loss is approached.

**Figure 12.**
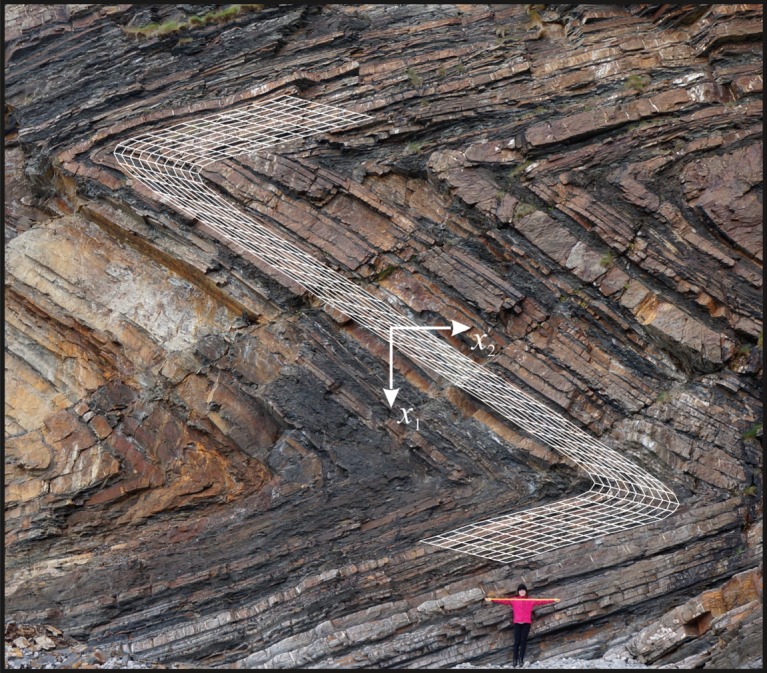
Deformation of an initially straight layer (shown in white) under the action of concentrated forces, superimposed on a detail of the photograph shown in figure 1*a*. The agreement between the deformed layer and chevron folding shows the possibility of describing this phenomenon through the loss of (E) of a constrained Cosserat material. (Online version in colour.)

Following Biot and assuming the two characteristic lengths to be equal, it is set $\ell =\tilde{\ell }=10$ cm, together with a null Poisson's ratio, *δ*=0 (assumed for simplicity). In the lack of precise evaluations, the orthogonal compliance ratios are set both to be small and equal to *α*=10^−2^ and *ε*=3×10^−6^. Accordingly, the ratio of the bending stiffnesses becomes *β*=10^−2^, so that the material approaches (E) loss. Four concentrated forces parallel to the *x*_2_-axis are placed within an infinite material: two forces with **P**=(0,±*P*) at points (±*d*_1_, ±*d*_2_), and two with **P**=(0,∓*P*/3) at points (±3*d*_1_, ±*d*_2_), where *d*_1_=35ℓ, *d*_2_=120ℓ and *P*=*c*_22_ℓ. The resulting system of forces is thus self-equilibrated. The deformation of an initially straight layer and of thickness 30ℓ (|*x*_2_|≤15ℓ) is superimposed on a detail of the photograph shown in figure 1*a*. The folded structure obtained from our results, shown in white in figure 12, evidences a good agreement with the actual deformation of the rock material (compare also with fig. 7 of [1]). It is noted that the key material parameters introduced to arrive at the folding structure shown in figure 12 are the ratios *β* and *ε*; on the other hand, it was found that the result is insensitive to variations in the ratios *α* and *δ*. Finally, it should be remarked that, due to the difficulties connected with the estimates of the material parameters, our result should only be taken as *indicative* of the possibility of describing chevron folding through a loss of (E) in a constrained Cosserat material. A more thorough investigation would be necessary to relate the folding to the actual material parameters of the rock formation.

## 9. Conclusion

Localized folding and faulting of an elastic continuum has been explained as a failure of ellipticity in a constrained Cosserat material, in a manner similar to that of the formation of shear bands in a plastic material. The adopted approach is perturbative and is based on the derivation of new Green's functions. Although the material employed in this study is an elastic solid with extreme anisotropy (and so an immediate application of the obtained results is to artificial materials with elements of ultra-contrasting stiffness), the constitutive tensor may represent the loading branch of an elastoplastic material, so that the obtained results are also pertinent to ductile materials deforming plastically and evidencing gradient effects. Chevron formations in rocks have been addressed with the proposed approach, so that structural folding can now be understood from a novel perspective.

## Footnotes

1 Within the nonlinear theory of elasticity, surface creasing or wrinkling on soft solids (such as elastomers or gels) can be described as a bifurcation problem [2–6]. Creasing of a surface is however different from the folding occurring within a bulk material addressed in the present article.

2 In both cases, the structure can be considered as an intercalation of stiff and compliant layers. In fact, when the tiles are simply superimposed on each other, they will be in contact at a discrete number of points, which creates a sort of weak layer.

3 The constitutive equations used in this article may represent the loading branch of an elastoplastic tangent operator, so that the assumed anisotropy can represent the result of an evolution due to loading, as in nonlinear elasticity or in elastoplasticity.

4 This situation shares analogies with the formation of shear bands in a solid, which formally occurs at the elliptic border, but can become visible near this border using a perturbative approach [15,32].

## Appendix A. Classical elasticity Green's function for an orthotropic solid

For the evaluation of the classical infinite-body Green's function, a procedure analogous to that employed in §3 is followed, setting *η*_1_=*η*_2_=0. In this case, the characteristic polynomial becomes

A 1 $$D^{cl}(\text{k})=c_{11}c_{66}k_{1}^{4}+[c_{11}c_{22}-c_{12}(c_{12}+2c_{66})]k_{1}^{2}k_{2}^{2}+c_{22}c_{66}k_{2}^{4}.$$

Note that $D^{cl}(\text{k})\equiv k^{4}\det{\text{A}}^{\left(C\right)}$, *k*=|**k**|. The *four* roots of the characteristic quartic polynomial can be either purely imaginary or complex conjugate and are placed symmetrically in the four quadrants of the *k*_2_-plane. In the classical elasticity case, the respective integrals (3.10) are evaluated in closed-form, yielding

A 2 $$u_{q}(x_{1},x_{2})=P_{p}G_{pq}(x_{1},x_{2}),\;(q=1,2),$$

with

A 3 $$\begin{array}{rl} G_{11}(x_{1},x_{2}) & =\frac{1}{2\pi p_{2}^{1/2}}\left[Y_{1}^{\left(1\right)}\;\text{Log}(z_{1})-Y_{2}^{\left(1\right)}\;\text{Log}(z_{2})\right],\\ G_{12}(x_{1},x_{2}) & =G_{21}(x_{1},x_{2})=-\frac{\left(c_{12}+c_{66}\right)}{2\pi p_{2}^{1/2}}(\theta_{1}-\theta_{2}),\\ G_{22}(x_{1},x_{2}) & =\frac{1}{2\pi p_{2}^{1/2}}\left[Y_{1}^{\left(2\right)}\;\text{Log}(z_{1})-Y_{2}^{\left(2\right)}\;\text{Log}(z_{2})\right] \end{array}\}$$

and

A 4 $$\begin{array}{rl} Y_{q}^{\left(1\right)} & =\frac{\left(c_{66}-c_{22}\rho_{q}^{2}\right)}{\rho_{q}},\;Y_{q}^{\left(2\right)}=\frac{\left(c_{11}-c_{66}\rho_{q}^{2}\right)}{\rho_{q}}\\ \text{and}\;z_{q} & ={\left(x_{1}^{2}+\rho_{q}^{2}x_{2}^{2}\right)}^{1/2},\;\theta_{q}={\text{Tan}}^{-1}\left(\rho_{q}^{-1}x_{1}x_{2}^{-1}\right), \end{array}\}$$

where

A 5 $$\begin{array}{rl} \rho_{1} & =-{\left(\frac{-p_{1}-p_{2}^{1/2}}{2c_{22}c_{66}}\right)}^{1/2},\;\rho_{2}=-{\left(\frac{-p_{1}+p_{2}^{1/2}}{2c_{22}c_{66}}\right)}^{1/2}\\ p_{1} & =c_{12}^{2}-c_{11}c_{22}+2c_{12}c_{66},\;p_{2}=\left(c_{11}c_{22}-c_{12}^{2}\right)\left(c_{11}c_{22}-{\left(c_{12}+2c_{66}\right)}^{2}\right). \end{array}\}$$

The closed-form result obtained above coincides with the corresponding result given by Watanabe [44] (note that in [44], p. 207, eqn. (7.1.10), the inner roots should read $\sqrt{\alpha \beta }$ and not $\sqrt{\beta \gamma }$).

In the special case where *c*_66_=0, the characteristic polynomial becomes $D^{cl}(\text{k})=\left(c_{11}c_{22}-c_{12}^{2}\right)k_{1}^{2}k_{2}^{2}$ and the formula (A2) does not apply since the characteristic polynomial has a double pole at $k_{2}^{\left(m\right)}=0$ in the *k*_2_-plane. Consequently, the Green's function then has to be evaluated in the distributional sense [45]. Resorting to the original double Fourier inversion integral, the following expressions for the displacement components can be obtained:

A 6 $$\begin{array}{rl} u_{1}(x_{1},x_{2}) & =-\frac{P_{1}c_{22}}{2\left(c_{11}c_{22}-c_{12}^{2}\right)}|x_{1}|\delta (x_{2})+\frac{P_{2}c_{12}}{4\left(c_{11}c_{22}-c_{12}^{2}\right)}\;\text{sgn}(x_{1})\;\text{sgn}(x_{2})\\ \text{and}\;u_{2}(x_{1},x_{2}) & =\frac{P_{1}c_{12}}{4\left(c_{11}c_{22}-c_{12}^{2}\right)}\;\text{sgn}(x_{1})\;\text{sgn}(x_{2})-\frac{P_{2}c_{11}}{2\left(c_{11}c_{22}-c_{12}^{2}\right)}|x_{2}|\delta (x_{1}), \end{array}\}$$

and the stress components can be evaluated in the sense of distributions as

A 7 $$\sigma_{11}(x_{1},x_{2})=-\frac{P_{1}}{2}\;\text{sgn}(x_{1})\delta (x_{2}),\;\sigma_{22}(x_{1},x_{2})=-\frac{P_{2}}{2}\;\text{sgn}(x_{2})\delta (x_{1}),\;\text{and}\;\sigma_{12}(x_{1},x_{2})=0,$$

where sgn () is the signum function. The equivalent von Mises stress in a classical material is defined then as $\sigma_{e}=\sqrt{3/2}∥\text{dev}\sigma ∥,$ which, in the special case where *c*_66_=0 and **P**=(0,−1), becomes $\sigma_{e}(x_{1},x_{2})=0.25\sqrt{3}\;\delta (x_{1}).$
